# Cold Microfiltration as an Enabler of Sustainable Dairy Protein Ingredient Innovation

**DOI:** 10.3390/foods10092091

**Published:** 2021-09-04

**Authors:** Thomas C. France, Alan L. Kelly, Shane V. Crowley, James A. O’Mahony

**Affiliations:** School of Food and Nutritional Sciences, University College Cork, T12 TP07 Cork, Ireland; 114345886@umail.ucc.ie (T.C.F.); a.kelly@ucc.ie (A.L.K.); shane.crowley@ucc.ie (S.V.C.)

**Keywords:** microfiltration, cold MF, partitioning, membrane fouling

## Abstract

Classically, microfiltration (0.1–0.5 µm) of bovine skim milk is performed at warm temperatures (45–55 °C), to produce micellar casein and milk-derived whey protein ingredients. Microfiltration at these temperatures is associated with high initial permeate flux and allows for the retention of the casein fraction, resulting in a whey protein fraction of high purity. Increasingly, however, the microfiltration of skim milk and other dairy streams at low temperatures (≤20 °C) is being used in the dairy industry. The trend towards cold filtration has arisen due to associated benefits of improved microbial quality and reduced fouling, allowing for extended processing times, improved product quality and opportunities for more sustainable processing. Performing microfiltration of skim milk at low temperatures also alters the protein profile and mineral composition of the resulting processing streams, allowing for the generation of new ingredients. However, the use of low processing temperatures is associated with high mechanical energy consumption to compensate for the increased viscosity, and thermal energy consumption for inline cooling, impacting the sustainability of the process. This review will examine the differences between warm and cold microfiltration in terms of membrane performance, partitioning of bovine milk constituents, microbial growth, ingredient innovation and process sustainability.

## 1. Introduction to Microfiltration

Membranes contain small pores of a defined average size, which permit molecules smaller than the pore size to pass through into the permeate stream, while larger molecules that cannot pass through the membrane are concentrated in the retentate. Membrane separation processes have numerous industrial applications, with the pressure-driven processes of microfiltration (MF), ultrafiltration (UF), nanofiltration (NF) and reverse osmosis (RO) being the most well established in the dairy industry with pore sizes of 10–0.1 µm, 0.10–0.01 µm, 10–0.1 nm and <0.1 nm, respectively [1]. Membrane filtration technology is widely used in dairy processing to concentrate, fractionate, isolate, demineralise, defat, and purify different target macro-molecules, such as proteins, phospholipids and lactose.

MF is a key unit operation in dairy processing, in which micrometre-scale particles (e.g., fat globules and bacterial spores) are retained while smaller molecules such as water, minerals, lactose and whey proteins permeate through the membrane. MF membranes cover a wide range of pore sizes, ranging from 0.1–10 µm, and are also associated with the lowest processing pressures (0.01–0.20 MPa) [1,2]. In comparison, higher operating pressures are required for UF, NF and RO with pressures ranging from 0.1–1.0, 1.5–3.0 and 3.0–5.0 MPa, respectively [1]. MF is increasingly being used in the dairy industry to reduce the bacterial load by removing bacteria cells (0.4–2.0 µm) and spores from skim milk prior to subsequent processing [3,4]. Based on the size difference between casein micelles (50–500 nm) and whey proteins (4–8 nm), MF technology is capable of fractionating the major proteins in milk in a chemical-free (e.g., no acids or bases required for pH adjustment) manner which is perceived as a “clean” approach when compared to alternative methods for protein fractionation (e.g., precipitation techniques). Thus, tight MF membranes (e.g., 0.1 µm) allow for the retention of casein micelles while serum proteins are removed, producing a casein-rich concentrate containing micelles in their native form, known as micellar casein concentrate (MCC), and a permeate enriched in whey proteins in their native form, known as ‘ideal’ or ‘native’ whey. This review will examine the effects of performing MF at low temperatures and how altering processing temperature ultimately affects membrane performance, fouling, protein and mineral partitioning, as well as microbial biodiversity and growth. The potential for ingredient innovation and the overall sustainability of the process are also considered. The studies cited throughout the review report the pasteurisation of feed material prior to filtration, with filtration operated under concentration mode, unless otherwise stated.

## 2. Choice of Membranes

There are a wide variety of MF membranes available to the dairy industry, differing both in the configuration and membrane construction material. Both the choice of membrane material and configuration depend on the intended application.

### 2.1. Membrane Structures and Configurations

**Figure 1:**
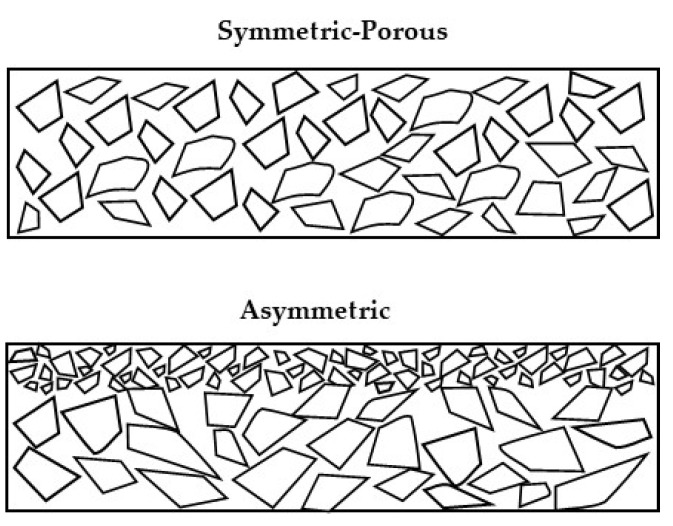
Schematic representation of the difference in structure of a symmetric (porous) and asymmetric membrane (modified from Smith [7], copyright 2013 Wiley-Blackwell).

The internal structure of MF membranes can be symmetric (isotropic) or asymmetric (anisotropic); isotropic membranes have pores of uniform size over the entire cross-section of the membrane, while anisotropic membranes have multiple layers, each with different structures and pore size (Figure 1) [5,6]. Due to the uniform structure of isotropic membranes, particles are often retained within the internal structure, resulting in reduced flux due to plugging of the pores [7]. Asymmetric membranes consist of a ‘skin’ layer on the surface of the membrane which acts as the actual selective barrier; below this thin ‘skin’ layer is a thicker porous sub-layer which has little effect on separation but provides mechanical support as well as allowing improved flux [7]. Asymmetric membranes are the most common commercially available membranes [8].

Membranes are available in hollow fibre, tubular, capillary and flat sheet geometries. Hollow fibre and tubular membranes have similar geometries, differing in terms of the tube diameters, with the former having outer diameters ranging from 0.05–0.5 mm while the latter have outer diameters of 5–25 mm [9]. For continuous membrane operations, these membranes are arranged into membrane elements/modules, with the type of module design depending on the membrane type (e.g., flat sheet membranes can be arranged into plate and frame configuration or spiral wound modules). There are four general categories of membrane configuration/design: spiral-wound, tubular, plate and frame and hollow fibre with both ceramic and polymeric materials available for use [8]. The choice of membrane module used depends on multiple factors, such as the capital costs associated with the module, resistance to fouling, ease of cleaning and the properties of the feed material (e.g., viscosity).

### 2.2. Choice of Membrane Material

The membrane material used in the MF of dairy streams is primarily polymeric, although ceramic materials are also used. There are a wide variety of organic (polymeric) and inorganic (ceramic) materials that can be used for the manufacture of membranes, although the choice of material used depends on the intended application, as each has specific advantages and disadvantages. Besides, the choice of membrane material, the cost associated with purchasing the membrane, cost of installation and operation, and the expected membrane lifetime are often the main factors determining membrane choice. The differences between polymeric and ceramic membranes in terms of milk protein fractionation has received some attention in recent years [10,11], with the efficiency of removal of serum proteins often being higher for ceramic membranes [12,13].

#### 2.2.1. Polymeric

The main polymers used to create MF membranes include polysulphone/polyethersulphone (PS/PES) and polyvinylidene fluoride (PVDF). PS and PES membranes have wide pH, temperature and chemical tolerances, as well as having the advantage of being able to be manufactured into a wide variety of configurations with a wide range of pore sizes. However, they are often limited to low operating pressures due to low mechanical strength. PVDF membranes possess many of the advantages of PES membranes, while they have narrower temperature and pH tolerances. Polymeric membranes have a broader pore size distribution compared to ceramic membranes and are relatively hydrophobic, which can facilitate protein adsorption [14]. The surface of these membranes can also be modified to alter the surface chemistry, modifying the way in which the membrane interacts with components of the feed system. Alternatively, hydrophobic polymers such as PVDF can be directly modified during the membrane production process to reduce hydrophobicity through the addition of hydrophilic polymers [15]. A hydrophilic membrane is most desirable in the processing of dairy streams to minimise binding of hydrophobic proteins to the membrane surface.

#### 2.2.2. Ceramic

Ceramic, also known as “inorganic”, membranes are formed by combining metals such as titanium or aluminium in different forms (e.g., oxides) with support materials (e.g., aluminium, stainless steel, silicon carbide). Generally, ceramic membranes are available in tubular form as either a single-channel tube or, more commonly, a multichannel element, with the latter design increasing packing density and mechanical strength. However, ceramic membranes have fewer disadvantages when compared to polymeric membranes. Due to the inorganic nature of the materials used to manufacture these membranes, they are inert to many chemicals and have wide temperature (>100 °C) and pH (0.5–13.5) tolerances [14]. As ceramic membranes can tolerate high temperatures, they can be sterilised, unlike polymeric membranes. However, ceramic membranes are both more brittle and heavier than their polymeric counterparts. In addition, the higher capital and running costs for ceramic membranes have made polymeric membranes an attractive alternative for milk processers. Inorganic membranes have longer life spans than polymeric membranes, with the life span of polymeric membranes depending on the frequency of use and the nature of cleaning (e.g., temperature at which cleaning is performed, type and strength of chemicals). Ceramic membranes also have the capability of allowing backflushing in which backpressure is applied to remove foulants from the surface of the membrane, helping to restore flux. In addition to this, to reduce fouling on ceramic MF membranes during filtration, a high cross-flow velocity can be used (5–7 m/s), which increases wall shear stress and therefore improves the efficiency of removal of loose foulant material [16].

## 3. Cold Microfiltration in Dairy Processing

Both MF and UF at low temperatures (i.e., <20 °C) have become increasingly common in the dairy industry due to evidence of reduced fouling, which may allow for longer run times [17,18,19], the opportunity to produce new ingredients with targeted protein profiles (i.e., β-casein-enriched ingredients), and reduced levels of denatured whey protein (i.e., higher levels of native whey protein) [20]. The differences between warm and cold MF in terms of operating conditions, membrane performance and partitioning are discussed below.

### 3.1. Temperature Range

Traditionally, both MF and UF of milk are performed at temperatures of 45–55 °C [21], with associated advantages of higher initial fluxes, resulting from lower viscosity and efficient removal of whey proteins [19,22]. However, the filtration of milk within this temperature range can cause issues with fouling and the growth of thermophilic bacteria. As a result, some dairy processors are transitioning from traditional warm MF (45–55 °C) towards cold MF. The term “cold”, in the dairy industry, when applied to MF (and also UF), covers a broad temperature range, with temperatures <20 °C generally being considered as low temperature filtration [23]. Selecting the temperature at which MF is performed is an important consideration, as it can have significant effects on membrane performance, the degree and type of fouling, partitioning of milk constituents and energy requirements of the plant, as well as the microbial quality of the ingredients produced.

### 3.2. Membrane Performance and Fouling

**Figure 2:**
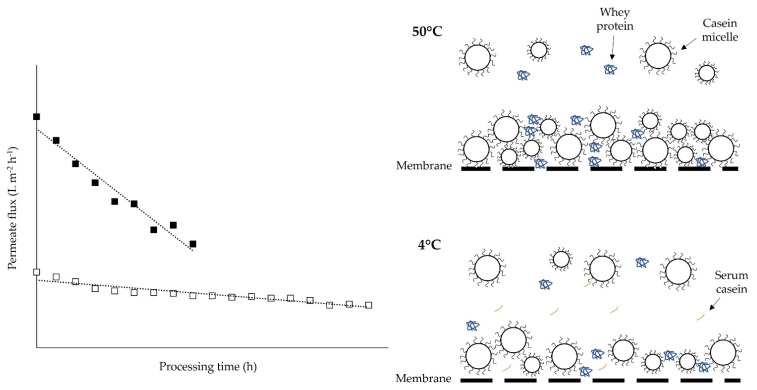
Schematic representation of permeate flux decline during warm (50 °C: ■) and cold (4 °C: □) microfiltration of skim milk (**left**; modified from [19], copyright 2017 International Dairy Journal) and differences in compactness of the deposit layer on the membrane surface after MF at the respective temperature (**right**).

Operating at low temperatures is associated with lower permeate flux due to higher viscosity of the permeate stream, as stated by Darcy’s law:

(1) $$J\;=\;\frac{\mathrm{TMP}}{\eta \;\times \;R\;}$$

where J is the permeate flux, TMP is the transmembrane pressure, η is the viscosity of the fluid permeating the membrane at a specific processing temperature and R is the overall hydraulic resistance consisting of resistance of the clean membrane, reversible and irreversible fouling. Thus, when performing filtration at cold temperatures, longer run times may be required to achieve the same volume concentration factor compared to operating at higher temperatures. One of the biggest challenges of membrane filtration is fouling, in which solutes accumulate at the membrane surface or within the membrane itself [24]. Although, during warm MF, initial flux levels are higher than for cold MF, fouling is more extensive with the former process [19]. McCarthy et al. [19] filtered unpasteurised skim milk using a 0.14-µm pore size ceramic membrane at 8.9 and 50 °C and reported that permeate flux was lower for cold MF compared to warm MF, although flux decreased rapidly during the warm MF process due to higher levels of fouling. Hartinger et al. [25] reported that MF of skim milk at 50 °C, under total recirculation mode, using a 0.1-µm spiral-wound PVDF membrane, resulted in a more rapid decline in flux and a more significant reduction in protein permeation throughout filtration, compared to MF at 10 °C. Similar findings have been reported for UF membranes, with Luo et al. [17] and Ng et al. [26] observing lower fouling at low processing temperatures (i.e., 10–15 °C) compared to higher temperatures (i.e., 50 °C). Barukčić et al. [27] measured water flux before and after MF of whey under total recirculation mode, using ceramic membranes with different pore sizes (0.1, 0.5 and 0.8 µm), at 20 and 50 °C, and reported that fouling intensity was lower at the lower processing temperature for each pore size. Therefore, even though initial flux levels are lower for cold MF (Figure 2), longer processing times with a more stable flux over time, as a result of lower fouling, could result in a reduced number of cleaning cycles and therefore reduced downtime during cleaning in place (CIP) and consumption of water and cleaning chemicals [28].

These studies all indicate that more extensive fouling occurs when filtration is performed at high processing temperatures (i.e., 50 °C) compared to low processing temperatures (i.e., 10 °C). Microfiltration of dairy streams at these high processing temperatures (45–55 °C) can also result in partial unfolding of whey proteins, particularly β-lactoglobulin, which can lead to more extensive fouling. β-lactoglobulin has a free cysteine group which becomes accessible as the protein unfolds. At temperatures up to 60 °C, reversible dissociation of β-lactoglobulin dimers to monomers and reversible unfolding occurs, resulting in increased sulfhydryl group reactivity [29], which can increase protein–protein interactions. Studies have shown that performing filtration at higher temperatures (i.e., 50 °C) results in a more compact fouling layer compared to low temperatures (i.e., 10 °C; Figure 2) [18,25,26]. Steinhauer et al. [18] reported that MF of β-lactoglobulin suspensions at 10 °C, with a 0.1-µm pore size, resulted in the formation of a loosely packed and highly permeable fouling layer; on the other hand, MF at temperatures ≥40 °C resulted in the formation of a thicker and more compact fouling layer with a higher fouling resistance, which was attributed to increased protein hydrophobicity and sulfhydryl group reactivity.

The differences in membrane performance and the extent of fouling at high and low temperatures is also evident when comparing permeate flux before and after increasing TMP. Hartinger et al. [25] examined the effect of a stepwise increase in TMP and the subsequent stepwise reduction in TMP on filtration resistance during the MF of skim milk at 10 and 50 °C. At 50 °C, a distinct hysteresis was observed in the flux data, indicating extensive accumulation of deposits on the membrane, whereas at 10 °C there was no distinct hysteresis in flux after the pressure cycle. The results suggest that the compression of the deposit layer at 10 °C is largely reversible, in contrast to the deposit layer formed at 50 °C. The high pressure, coupled with the high processing temperature, may allow for increased protein–protein interactions and crosslinking of foulants, resulting in a more compressed fouling layer than that generated at lower processing temperatures. Thus, although warm MF is associated with higher initial fluxes, extensive fouling can reduce membrane life and alter filtration performance, while cold MF can provide stable flux with less extensive fouling, albeit with lower flux.

### 3.3. Composition of the Fouling Layer

During the MF of skim milk and other dairy streams (e.g., whey), fouling occurs, resulting in the formation of a cake layer varying in composition, with proteins and minerals (e.g., calcium phosphate) being the principal fouling materials [5,24,30,31]. Protein fouling is caused by the deposition and adsorption of proteins (whey proteins and casein) onto the surface of the membrane or within the pores of the membrane [31,32], while mineral fouling is largely due to calcium, facilitating protein–protein and protein-membrane interactions. Tan et al. [24] found both whey protein and casein to be the primary foulants during cold MF of raw skim milk using a 1.4 µm ceramic membrane, while the contribution of minerals to membrane fouling was minor. The solubility of calcium phosphate is inversely proportional to temperature; thus, calcium phosphate deposition/scaling on the membrane surface or within pores during cold MF is likely to be less significant than during warm MF. Extensive membrane fouling by proteins during MF not only reduces the permeation of proteins through the membrane but is also associated with reduced membrane selectivity, reduced yield, and increased membrane cleaning times [25,33].

### 3.4. Microbial Impact

**Figure 3:**
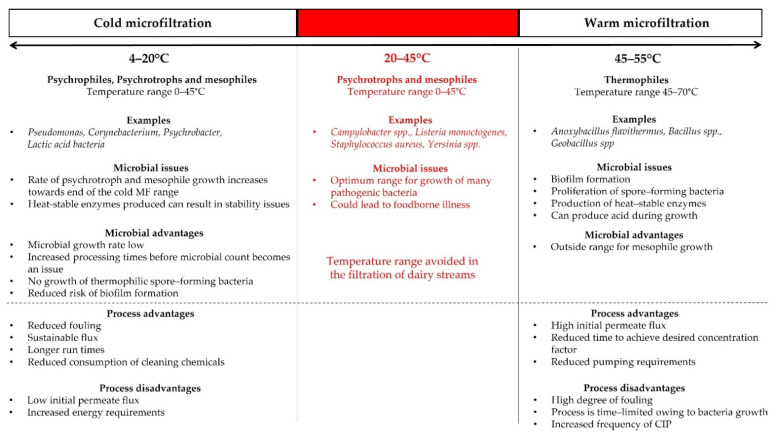
Schematic representation of some of the microbial considerations when selecting microfiltration processing temperature and the implications of such processing temperatures for processing efficiency and finished product quality. Information regarding examples of bacteria species sourced from [36,37,38,39].

MF with nominal pore sizes of 0.8–1.4 µm can be used to reduce the microbial load of milk, such as in the production of extended shelf-life milk [34] and in the production of infant formula-grade milk powders [35]. It also provides the dairy industry with MF permeate with improved microbial quality prior to the generation of milk-derived protein ingredients. However, if this is not performed, and membranes with pore sizes of 0.1 µm are employed in the fractionation of milk proteins, bacteria present are retained and concentrated in the retentate. In the dairy industry, MF systems are operated in continuous mode for long periods of time, which can result in the accumulation of microorganisms and support the formation of biofouling on the MF membrane. Filtration in the dairy industry is often performed at temperatures suitable for the growth of microorganisms; in addition to this, high TMP, lack of mechanical cleaning and constant flow of feed through the membrane make membranes susceptible to biofilm formation. Therefore, the temperature at which filtration is performed is an important consideration for processors, as it influences microbial growth and the bacterial community that can grow (Figure 3).

In order to delay and/or limit biofilm formation and microbial growth, the temperature at which MF is performed can be reduced. Filtration at high temperatures (i.e., 45–55 °C) can provide optimum conditions for the growth of thermophilic bacteria, which can proliferate at temperatures between 45–70 °C [36,37]. Common thermophilic bacteria such as *Anoxybacillus flavithermus*, *Bacillus* spp. and *Geobacillus* spp. can produce heat-stable proteases and lipases, with important implications for the quality of dairy products; their spore-forming and biofilm-forming abilities also present challenges, as they are difficult to remove and/or inactivate and are also capable of producing acid on growth, reducing pH [28,36,38]. Studies have indicated that operating at high temperatures can result in a faster rate of microbial growth and biofilm formation than at low temperatures. Chamberland et al. [39] reported that the rate of biofilm formation on UF membranes during skim milk processing at 15 °C was slower than at 50 °C. These authors found that bacterial growth was significantly higher at 50 °C, with the number of 16S rRNA gene copies on the membrane increasing from 3.21 ± 0.12 to 8.83 ± 1.58 log_10_ gene copies per cm^2^ after 15 h of processing, while, at 15 °C, the number of gene copies on the membrane only increased from 3.31 ± 0.24 to 3.86 ± 0.58 gene copies per cm^2^ after 48 h of filtration.

During filtration of milk at low temperatures (<15 °C), psychrophiles and psychrotrophic bacterial genera, such as *Pseudomonas*, *Psychrobacter* and *Corynebacterium* are capable of growing [39]. Facultative psychrotrophs can grow within a broad temperature range (0–40 °C), occurring at higher numbers in milk compared to obligate psychrophiles (0–15 °C) [37]. The optimal growth temperature for most psychrotrophic bacteria is between 20–30 °C; therefore, the extent of proliferation during cold MF is low [40]. Operating at the upper end of the cold MF range can result in the growth of some mesophilic organisms [37]. However, the growth rate of such mesophilic bacteria (e.g., lactic acid bacteria) is low at these temperatures (i.e., 15 °C). As these higher temperatures (15–20 °C) facilitate the growth of mesophilic bacteria and are close to the optimal metabolic activity of psychrotrophic bacteria, such temperatures are generally avoided for MF in dairy processing. Schiffer and Kulozik [28] examined the effect of temperature (10, 14, 16, 20 °C and 55 °C) on microbial growth during MF of skim milk under feed-and-bleed mode (permeate and 10 L h^−1^ of retentate was continuously removed during MF and replaced with skim milk), using a 0.1 µm PES membrane; the authors reported that filtration at 55 °C was deliberately stopped after 10 h due to a sudden drop in pH, attributing this to microbial activity. Within the cold MF range, microbial growth was highest at 20 °C, followed by 16 °C and 14 °C taking 8, 10 and 17 h, respectively, to reach the critical colony forming unit level of 10^5^ cfu mL^−1^; at 10 °C, microbial counts remained almost constant over 24 h of MF. The bacterial communities responsible for the increasing microbial counts at 16 and 20 °C in the aforementioned study were not determined, and so it was not clear whether mesophilic bacteria made a considerable contribution towards microbial counts at the upper end of the cold MF range.

In order to improve the microbial quality of ingredients produced using filtration, there has been a trend towards cold MF, as the rate of microbial growth and biofilm formation is lower than that of warm MF. Therefore, performing MF at low temperatures (i.e., ≤10 °C) may allow for longer run times before microbial growth becomes an issue for hygiene standards. However, reducing the temperature at which filtration is performed results in reduced flux and can also increase energy demands. Therefore, research on finding the optimum temperature for maintaining microbial growth and biofilm formation low while also optimising filtration performance (e.g., permeate flux) is still required.

### 3.5. Protein Partitioning

During the MF of dairy streams, a cake layer is formed, which results in the membrane becoming less permeable; this fouling layer is known to act as a secondary membrane, altering the permeate flux and the selectivity of the membrane. There have been numerous studies comparing the effect of temperature, as well as differences between ceramic and polymeric membranes, on the efficiency of protein fractionation [11,12,18,25,41]. Hartinger et al. [25] studied the influence of temperature and TMP on protein fractionation during MF of skim milk using a 0.1-µm membrane. The authors reported that, at 50 °C, the initial rate of reduction in protein permeation was higher than that at 10 °C, with MF at 50 °C also being associated with a higher mean reduction in the permeation of protein (2.8% h^−1^ vs. 1.2% h^−1^ at 50 and 10 °C, respectively) between 40 and 180 min of MF; this was attributed to a more extensively fouled membrane and temperature-induced aging of the deposit layer at 50 °C.

Although many of these studies have examined the effects of temperature on protein partitioning at either one temperature or at two different temperatures (often involving a comparison between low and high temperatures), there is limited information available on the effect of different temperatures within the cold MF range on protein partitioning and fouling. Jarto et al. [41] reported that MF of skim milk at 13 °C resulted in higher whey protein permeation compared to MF at 5 °C. Similar results were reported by France et al. [33], who observed that MF of skim milk at 12 °C resulted in higher concentrations of β-lactoglobulin and α-lactalbumin in permeate, compared to at 4 and 8 °C, although differences between temperatures were not significant. Thus, temperature affects protein partitioning during filtration primarily through differences in the form and extent of fouling. These studies indicate that processing temperature influences whey protein permeation. MF of milk at low temperature (i.e., 4 °C) also results in the permeation of casein, in particular β-casein, through the membrane and into the permeate, resulting in the generation of a β-casein-enriched whey, a phenomenon that is not associated with MF performed warm. Therefore, not only does the temperature at which MF is performed influence whey protein permeation, but also the protein profile of the resulting permeate and retentate.

The choice of membrane material can also impact the efficiency of whey protein permeation and the composition of the final permeate and retentate streams. Zulewska et al. [12] examined the efficiency of serum protein removal from the MF of skim milk at 50 °C, using both spiral-wound polymeric (0.3 µm) and two ceramic membranes (uniform transmembrane pressure and graded permeability MF systems) of equivalent pore size (0.1 µm). The authors reported that permeate generated using ceramic membranes had a higher concentration of protein than that generated using the polymeric membrane. The use of ceramic MF membranes resulted in higher levels of β-lactoglobulin in the permeates, with an overall higher efficiency of whey protein removal than the spiral-wound polymeric membrane (64.4–61.0% vs. 38.6%). The authors suggested that the difference observed were, in part, due to the hydrophilic nature of ceramic membranes resulting in lower protein adsorption to the membrane when compared to the hydrophobic polymeric membrane. Similar results have since been reported by Carter et al. [13] and Beckman et al. [42].

### 3.6. Mineral Partitioning

Performing MF at low temperatures changes the composition of final permeate, not just in terms of the protein profile, but also of the mineral profile. As temperature decreases, colloidal calcium phosphate (CCP) within casein micelles solubilises, increasing the concentration of calcium and other minerals in the serum phase [43]. Therefore, when MF is performed at low temperatures (i.e., 4 °C), there is a higher concentration of soluble minerals (e.g., calcium) that can permeate the membrane, resulting in a higher concentration of ash in the permeate [44]. There have been numerous studies detailing the effect of processing temperature on the partitioning of calcium during both UF and MF of skim milk; the authors of these studies report that reducing the temperature at which filtration is performed increases the concentration of total and/or ionic calcium in the permeate [17,44,45,46]. Crowley et al. [47] analysed MCC powder produced by MF of skim milk at low (<10 °C) or high temperature (50 °C) and reported that the MCC powder generated at cold temperatures had lower levels of calcium and phosphorus compared to that made from warm MF, while levels of monovalent ions were relatively unchanged. The authors also studied the effect of MF on the rehydration characteristics of MCC powders and reported that MCC generated from cold MF had superior dispersion characteristics, with 50–60% less sedimentation following centrifugation; this was partially attributed to lower Ca content.

The presence of high concentrations of calcium in β-casein-enriched whey following cold MF (0.1–0.5 µm) of skim milk can lead to challenges with subsequent downstream processing of such novel whey streams. On warming the β-casein-enriched whey, the strength of hydrophobic interactions increases, causing the self-association of molecular β-casein (7–8 nm) and an increase in particle size, ultimately resulting in the formation of β-casein micelles (20–30 nm); this phenomenon is normally reversible on cooling, with β-casein returning to a monomeric state [48,49,50]. However, in the presence of calcium (e.g., 8–15 mM at 37 °C), these β-casein micelles can aggregate via calcium-mediated crosslinking, resulting in a particle size of >1 µm, and leading to precipitation of the aggregated β-casein [48,51]. Therefore, following cold MF of skim milk, a demineralisation step is often applied to the permeate stream to reduce the concentration of soluble minerals, allowing for stable downstream processing or indeed allowing for the controlled, reversible, thermal aggregation of β-casein without incurring precipitation [20]. Thus, MF of skim milk at low temperatures results in increased concentrations of total divalent cations (e.g., calcium) in the permeate, altering the mineral composition of both the permeate and retentate compared to that produced from warm MF, which can have important implications for ingredient functionality.

### 3.7. Enzyme Partitioning

The principal indigenous milk proteinase, plasmin, can result in challenges for the proteolytic stability of filtration-derived milk protein ingredients. During warm MF of milk using a 0.1-µm membrane, casein micelles are concentrated in the retentate, while whey proteins are removed in the permeate, with such permeate containing many inhibitors of the plasmin system, including β-lactoglobulin. As plasmin is primarily associated with casein micelles through lysine binding and to a lesser extent through electrostatic interactions [52,53], it becomes concentrated in the retentate. Consequently, plasmin activity in the retentate increases, with subsequent diafiltration resulting in further increases in plasmin activity as inhibitors are further removed [54].

During cold MF, β-casein dissociates from the casein micelles into the serum phase, where it passes through the membrane into the permeate stream. Therefore, there remains the question whether temperature-induced changes in casein micelles during cold MF also affects the association of plasmin with the casein micelles. As the principal substrate for plasmin is β-casein, plasmin activity in such permeates may lead to issues with β-casein hydrolysis on further downstream processing and in products incorporating the ingredient. France et al. [44] studied the effect of temperature (4, 8, 12, 16 or 20 °C) on enzyme partitioning during MF of skim milk under total recirculation mode and reported that temperature had no significant effect on plasmin activity in β-casein-enriched permeate; therefore, the effect of temperature on plasmin activity in permeates is not as pronounced as that seen for β-casein. Furthermore, in the purification of β-casein, the serum phase containing many of the inhibitors are removed; thus, in the absence of such inhibitors, plasmin activity may be even greater. The presence of plasmin in permeate streams containing β-casein could result in negative consequences, with plasmin-induced hydrolysis of β-casein potentially reducing levels of intact β-casein, thus reducing its functional properties of value in food formulations.

## 4. Microfiltration as an Enabler of Dairy Ingredient Innovation

MF of skim milk allows for the generation of specific ingredients, with the temperature at which MF is performed strongly influencing the composition and, therefore, the functionality and applications of the ingredients generated, as discussed below.

### 4.1. Warm Microfiltration Applications in the Dairy Industry

MF of dairy streams has many different applications depending on the feed material, processing temperature, and pore size. One of the first applications of warm MF in the dairy industry was the removal of residual fat and denatured whey proteins from whey in the production of whey protein isolate (WPI). MF membranes with a pore size of less than 1.0 µm retain any residual fat (i.e., that from the cheesemaking process in sweet whey), phospholipids, and denatured protein, thereby allowing for a higher protein content to be achieved in the final WPI product. This MF process creates a co-product with a high content of protein, fat and phospholipid which can be further processed to produce a whey protein phospholipid concentrate (WPPC). A standard for the composition of WPPC was established in 2015 by the American Dairy Products Institute which state that such products must have a minimum of 50% protein on a dry basis, minimum 12% fat, maximum of 8% ash and maximum of 6% moisture. WPPC is a functional dairy ingredient that can add functionality to ingredients at a low cost (e.g., in cakes as an egg replacement) [55], with its biggest potential use being in nutritional applications due to its rich phospholipid profile. Although MF for WPI production has been traditionally performed at 45–55 °C, transitioning to cold processes could allow for reduced fouling and higher native whey protein levels.

Warm MF in the dairy industry has also been used for the removal of bacteria from milk using a typical pore size of ~1.4 µm [2,56]. Here, the objective is to remove bacterial cells, somatic cells and endospores, while minimising the retention of proteins to maintain the composition of the MF permeate as close to that of the milk feed. Therefore, 1.4-µm membranes are most widely used, allowing for the right balance between high flux, bacteria removal and the permeation of other milk components with little or no rejection. A reduction in the pore size (e.g., 0.5–0.8 µm) of MF membranes results in improved reduction in bacteria but can lead to a more rapid decline in flux and increased retention of casein micelles [57]. In the dairy industry, the removal of bacteria is primarily performed at high temperatures (i.e., 50 °C). Such temperatures allow for the growth of thermophilic spore-formers and can lead to the formation of resistant biofilm formation, while using low-temperature MF can reduce the risk of bacterial growth and proliferation of thermophilic spore-formers. Studies have shown that cold MF of skim milk using ceramic membranes effectively reduces bacterial cells and somatic cells [4,58,59]; therefore, cold MF can be used to reduce bacterial load and somatic cell count while reducing the risk of thermophilic bacterial growth and biofilm formation, which could reduce final product quality. Within the dairy industry, cold MF is now also used to improve the microbial quality of the feed material prior to the production of ingredients, e.g., Micelate Prestige^TM^, a product currently produced by FrieslandCampina, is a cold-processed, native micellar casein isolate (MCI) which has undergone cold MF to improve the microbial quality of the ingredient.

Following MF of whole milk using a 1.4-µm membrane, as described above, fat globules (0.1–15 µm) are also retained in the retentate. The milk fat globule membrane (MFGM) retained in this MF retentate can be isolated to produce MFGM material. A recent study reported that MF of raw milk resulted in the production of high purity MFGM material [60]. As MFGM material is often isolated from heavily processed dairy streams (e.g., buttermilk and high fat retentate generated following MF of WPC), the process mentioned offers the dairy industry a practical method to produce MFGM material from a less heavily processed stream.

**Figure 4:**
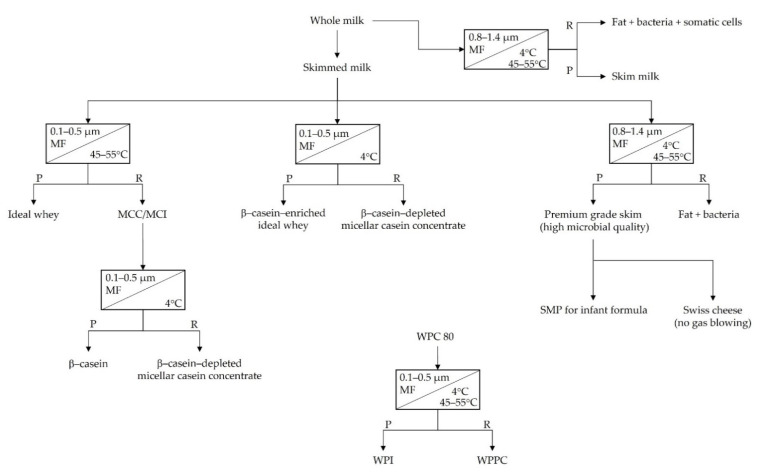
Schematic representation of the use of microfiltration (MF) to improve microbiological quality and generation of different milk protein-derived ingredients as influenced by processing temperature. MCC, micellar casein concentrate; MCI, micellar casein isolate; SMP, skim milk powder; WPC, whey protein concentrate; WPI, whey protein isolate; WPPC, whey protein phospholipid concentrate; P, permeate; R, retentate.

Warm MF of skim milk, when performed with a 0.1-µm pore size membrane, results in two streams, a casein micelle-enriched retentate and a whey protein-enriched permeate (Figure 4). Performing MF at these high temperatures ensures that β-casein does not dissociate from the micelle and into the serum phase as hydrophobic interactions remain strong. The permeate produced from warm MF of milk is often referred to as ‘ideal’ or ‘native’ whey, with a composition similar to that of sweet whey but is free of many of the contaminants present in sweet whey, such as glycomacropeptide, starter culture, colour residues, lipid material, lactic acid and coagulant. In addition, ideal whey has lower fat content, improved protein quality and higher levels of native whey proteins when compared to sweet whey, as sweet whey is typically subjected to more intense thermal treatment [61,62]. The retentate stream or co-product following diafiltration (UF permeate or RO water) is known as MCC and is enriched in native micellar casein. The production of MCC has multiple applications in nutritional and clinical products and in sports nutrition products, providing a slow release of amino acids [63]. MCC can also be used as a starting feed material in the production of β-casein. Warm MF of cheese milk is also used in the manufacture of cheese, enhancing cheese yield and output. MF instead of UF also provides the added benefit of depleting most of the serum proteins prior to reaching the cheese vat and are therefore the whey is not contaminated with colour residues (e.g., annatto) and other residues from the cheese making process [64].

### 4.2. Cold Microfiltration

There has been an increasing trend in the use of cold MF in the dairy industry. One of the opportunities cold MF offers is the production of new ingredients with selected protein profiles (e.g., β-casein ingredients).

#### 4.2.1. Influence of Temperature on β-Casein Dissociation

The adoption of cold MF offers dairy processors the opportunity to produce ingredients from skim milk with differentiated protein profiles. Of the four caseins (α_s1_, α_s2_, β and κ), β-casein enrichment/isolation is the most viable and widely practised. Bovine β-casein is held within the casein micelle primarily via hydrophobic interactions [65]. Reducing temperature results in a decrease in the strength of hydrophobic interactions, resulting in the dissociation of β-casein from the interior to the surface of the micelle, from where it dissociates into the serum phase of milk [66]. At low temperature (i.e., ≤4 °C), β-casein primarily exists in a monomeric state [67,68], although even at these temperatures polymeric structures are not entirely absent [69]. This phenomenon can be exploited in the production of β-casein-enriched whey during cold MF of milk, because at these low temperatures (i.e., ≤4 °C), monomeric β-casein can permeate through the MF membrane, producing a β-casein-enriched permeate and a β-casein-depleted retentate (Figure 4).

The temperature at which filtration is performed at within the cold MF range has a significant impact on serum β-casein concentration, with increasing processing temperature significantly reducing β-casein concentration in the permeate stream. A study by France et al. [44] reported that increasing processing temperature in the range 4–20 °C during the cold MF of skim milk, under total recirculation mode, reduced the concentration of β-casein in the permeate stream, from 2.02 to 0.53 mg/mL at 4 and 16 °C, respectively, under sub-critical flux conditions. By controlling temperature, the strength of hydrophobic interactions can be manipulated, ultimately influencing the concentration of β-casein in the serum phase that can permeate across the membrane.

The complete retention of caseins is often desired in milk protein fractionation to obtain a whey protein stream free from serum casein. Therefore, the increasing trend towards cold MF presents challenges to dairy processors in the production of native whey, as the purity of the whey protein fraction is affected. A recent study by Schiffer et al. [70] examined the effect of added calcium and pH on milk protein fractionation during the MF of skim milk, under total recirculation mode, using a 0.1-µm ceramic membrane at different temperatures within the cold MF range (10–20 °C). The authors hypothesised, and the results confirmed, that the addition of calcium to skim milk prior to cold MF would reduce casein concentration in the permeate as it has been shown that calcium concentration influences the ratio of soluble and micellar casein, partly by increasing milk protein hydrophobicity [71,72], and thereby reducing the amount of casein in the permeate. At 10 and 14 °C, the strongest effect for β-casein was observed at a level of 5 mM calcium and above; however, addition of >5 mM calcium negatively affected membrane performance and resulted in enhanced deposit formation on the membrane, as well as reducing whey protein permeation.

#### 4.2.2. Production of β-Casein-Enriched Ingredients via Cold Microfiltration

There are different approaches that can be used to generate β-casein and β-casein-enriched whey. There have been numerous studies investigating the permeation of β-casein during cold MF of different feed materials [20,73,74]. The most common starting material for the generation of β-casein-enriched whey is skim milk; however, other materials/ingredients can also be used, such as whole milk, MCC and sodium caseinate, although the latter is rarely used.

There are two common MF approaches that can be utilized in the manufacture of β-casein (Figure 4). One approach is referred to as ‘cold-then-warm’ MF, in which skim milk is first subjected to cold MF often holding at ≤4 °C to facilitate, and maximise, the release of β-casein into the serum phase. Most commonly, a 0.1-µm membrane is used, allowing the serum proteins and serum β-casein to permeate the membrane while the casein micelles are retained, generating a β-casein-depleted retentate [20]. The β-casein-enriched whey can be used as an ingredient without further fractionation, or it can undergo further processing to produce β-casein concentrate or isolate [75]. The β-casein-enriched whey can be ultrafiltered and diafiltered to further enrich and demineralise the permeate; for example, O’Mahony et al. [20] achieved this at low temperature (i.e., 1–6 °C) using a 10-kDa molecular weight cut-off membrane and chilled water as the diafiltration media. As previously mentioned, the removal of calcium and other soluble minerals prevents extensive calcium-mediated crosslinking of β-casein and subsequent precipitation. Following this, the concentrated and demineralised permeate is heated (25–50 °C) to increase hydrophobic interactions, resulting in the controlled self-association of β-casein and an increase in particle size [50]. O’Mahony et al. [20] warmed the permeate to 25 °C and passed it through a 0.5-µm polymeric membrane, resulting in the removal of serum proteins while the micellised β-casein was retained, yielding two streams, a β-casein concentrate retentate and an ‘ideal’ whey permeate.

The second approach can be referred to as ‘warm-then-cold’ MF, wherein warm MF (~45–55 °C; 0.1–0.5 µm) of the skim milk is performed, removing the serum proteins (permeate) while retaining the casein micelles (retentate) and the β-casein due to hydrophobic interactions within the micelles. Following this, cold MF (i.e., 4 °C) of the micellar casein retentate is performed, yielding a β-casein-depleted MCC retentate and a β-casein permeate. The application of membrane filtration allows for the production of high purity β-casein (up to 96% of total casein); however, yields are typically low (≤20%), although yields can be increased through additional β-casein dissociation and recovery of the β-casein depleted retentate [76].

If the feed material for cold MF is MCC rather than skim milk, a β-casein permeate relatively free of serum proteins can be obtained. Schäfer et al. [74] microfiltered MCC at ≤5 °C using a 0.1-µm pore size ceramic membrane, heated the β-casein permeate to 50 °C to achieve self-association of the β-casein, and then subjected the heated permeate to UF (10 kDa) to achieve further enrichment of β-casein. As the original permeate contained little or no whey proteins, an additional MF step to remove whey proteins was not required. Thus, cold MF can be exploited by dairy processors to generate innovative ingredients with tailored protein profiles when feed material containing casein is used.

Polymeric membranes such as PVDF, polyacrylonitrile (PAN) or PES are commonly used for the fractionation of caseins. Crowley et al. [23] studied the processing and protein fractionation characteristics of different polymeric membranes during cold MF of skim milk. The authors reported that PES and PVDF membranes, with equivalent pore size, had essentially identical selectivity; however, the PES membrane resulted in higher flux and lower fouling.

## 5. Sustainability

The sustainability of a process must consider its effects on environmental, social, economic, and technical aspects with capital and operational costs, consumption of energy, footprints, consumption of chemicals, product loss, valorisation of co-products and membrane fouling some examples of the various criteria that must be considered when assessing the sustainability of membrane processing [77,78]. In terms of protein fractionation, MF creates a green image for the process and the ingredients generated, as alternative techniques involve the addition of chemicals (e.g., pH adjustment) or the generation of large quantities of waste buffer (e.g., chromatography). Considering the factors mentioned and the increasing demand for more sustainable dairy processing, cold MF offers both opportunities and challenges in meeting such requirements. Maximising the active production time of a membrane filtration plant reduces the frequency of cleaning and reduces the consumption of cleaning chemicals and the associated environmental impact. As mentioned throughout this review, cold MF of skim milk offers increased processing times due to reduced fouling and a slower rate of decline in permeate flux. Schiffer and Kulozik [28] used the data obtained from their study regarding flux stability, pH, and bacterial counts at different temperatures (10, 14, 16, 20 and 55 °C) during the MF of skim milk to approximate the number of production cycles and overall annual process times. The authors reported that possible production times were highest for MF at 10 °C (>24 h), while operation for only 7 h was possible for MF at 55 °C; thus, the number of annual production cycles and therefore the number of cleaning cycles was calculated to decrease with decreasing processing temperature. The study highlights that, even though permeate flux at low temperatures is not comparable to that at higher temperatures, the improved microbial quality and sustainable flux enables longer run times and reduced cleaning cycles. Reducing the number of CIP cycles reduces the quantity of water and cleaning agents consumed, reducing the impact of the process on the environment, resulting in a more sustainable process. In addition to this, other factors such as product loss resulting from mixing of product and cleaning solutions and energy requirements need to be considered.

The extent and type of fouling also affects the severity of cleaning required to restore membrane performance to acceptable limits. As discussed, performing MF at high temperatures can result in extensive fouling, as well as the formation of biofilms produced by thermophilic bacteria such as *Bacillus* spp., which have been shown to be one of the species most resistant to CIP solutions [79]. Therefore, cold MF of dairy streams may result in shorter cleaning times and use of reduced amounts or strengths of cleaning agents, ultimately reducing the environmental impact of the process, as a more loosely packed fouling layer is produced.

Another important consideration in the sustainability of a process is the energy requirement. One of the factors influencing the mechanical and thermal energy requirements during membrane filtration is the temperature at which filtration is performed. As the temperature of skim milk is decreased, the apparent viscosity increases, which increases the pumping energy requirements for feed and retentate recirculation. Méthot-Hains et al. [80] reported that UF of skim milk required to reach a volume concentration factor of 3.6 at 10 °C resulted in a higher total energy consumption compared to at 50 °C; the authors also reported that, at 10 °C, pumping energy was 2.3 times higher than at 50 °C, which can be attributed to the higher viscosity of the feed and retentate during filtration at low temperatures. France et al. [33] studied the effect of temperature on the mechanical and thermal energy consumption during cold MF of skim milk, and reported that operating at 4 °C resulted in considerably higher (~10.6%) mechanical and thermal energy requirements compared to 12 °C. Therefore, although cold MF offers opportunities to improve the sustainability of the process through reduced CIP cycles and therefore reduced consumption of chemicals and water as a result of reduced protein and bio-fouling, increased electrical input will contribute to increased operational costs.

## 6. Conclusions

MF has become an integral unit operation within the dairy industry, with the emergence of cold MF driven by increased demand for novel ingredients with enhanced functional properties, improved microbial quality, and improved membrane performance. However, a better understanding of the effect of temperature on deposit formation on membranes, in particular different temperatures within the cold MF range, which can ultimately lead to improved protein permeation and flux, is required. Cold MF can result in improved microbiological quality, longer, more sustainable, processing times and reduced fouling, extending the life span of membranes as well as providing dairy processors opportunities to produce new functional milk-derived protein ingredients, all acting as an incentive to shift from warm to cold MF. However, although increased processing times and reduced frequency of CIP can be achieved through cold MF, high operational costs associated with higher energy consumption, means research is required to reduce the energy consumption of the process. Recent literature reports promising concepts for improving the MF of dairy streams, such as developments in surface modification (e.g., charged membranes), offering the potential to improve issues relating to fouling. For the MF of dairy streams, these and other approaches will form a future role in improving efficiency and therefore the sustainability of cold MF in dairy processing.

## Data Availability

Data sharing is not applicable to this article.
